# The utility of P53 immunohistochemistry in the diagnosis of Barrett's oesophagus with indefinite for dysplasia

**DOI:** 10.1111/his.14642

**Published:** 2022-05-20

**Authors:** Wladyslaw Januszewicz, Nastazja D Pilonis, Tarek Sawas, Richard Phillips, Maria O'Donovan, Ahmad Miremadi, Shalini Malhotra, Monika Tripathi, Adrienn Blasko, David A Katzka, Rebecca C Fitzgerald, Massimiliano di Pietro

**Affiliations:** ^1^ Early Cancer Institute Department of Oncology University of Cambridge Cambridge UK; ^2^ Department of Gastroenterology Hepatology and Clinical Oncology, Centre of Postgraduate Medical Education Warsaw Poland; ^3^ Mayo Clinic Rochester Minnesota USA; ^4^ Department of Histopathology, Addenbrooke's Hospital Cambridge UK

**Keywords:** biomarkers, clinical pathology, oesophageal neoplasms, observer variations

## Abstract

**Aims:**

Barrett's oesophagus with indefinite for dysplasia (BE‐IND) is a subjective diagnosis with a low interobserver agreement (IOA) among pathologists and uncertain clinical implications. This study aimed to assess the utility of p53 immunohistochemistry (p53‐IHC) in assessing BE‐IND specimens.

**Methods and results:**

Archive endoscopic biopsies with a BE‐IND diagnosis from two academic centres were analysed. First, haematoxylin and eosin‐stained slides (H&E) were reviewed by four expert gastrointestinal (GI) pathologists allocated into two groups (A and B). After a washout period of at least 8 weeks, H&E slides were reassessed side‐to‐side with p53‐IHC available. We compared the rate of changed diagnosis and the IOA for all BE grades before and after p53‐IHC. We included 216 BE‐IND specimens from 185 patients, 44.0 and 32.9% of which were confirmed after H&E slide revision by groups A and B, respectively. More than half the cases were reclassified to a non‐dysplastic BE (NDBE), while 5.6% of cases in group A and 7.4% in group B were reclassified to definite dysplasia. The IOA for NDBE, BE‐IND, low‐grade dysplasia (LGD) and high‐grade dysplasia (HGD)/intramucosal cancer (IMC) was 0.31, 0.21, −0.03 and −0.02, respectively. Use of p53‐IHC led to a >40% reduction in BE‐IND diagnoses (*P* < 0.001) and increased IOA for all BE grades [κ = 0.46 (NDBE), 0.26 (BE‐IND), 0.49 (LGD), 0.35 (HGD/IMC)]. An aberrant p53‐IHC pattern significantly increased the likelihood of reclassifying BE‐IND to definite dysplasia (odds ratio = 44.3, 95% confidence interval = 18.8–113.0).

**Conclusion:**

P53‐IHC reduces the rate of BE‐IND diagnoses and improves the IOA among pathologists when reporting BE with equivocal epithelial changes.

## Graphical abstract

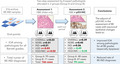

## Introduction

Barrett's oesophagus (BE) is an acquired metaplastic condition of the oesophagus, which develops in the context of chronic gastro‐oesophageal acid reflux.^1^ BE is a premalignant condition to oesophageal adenocarcinoma (EAC), with a progression rate of approximately 0.3% per year.^2^ Therefore, patients with BE are recommended to undergo endoscopic surveillance to diagnose neoplasia at early stages, which can be cured endoscopically.^3^, ^4^ The presence of dysplasia remains the strongest risk factor for cancer progression and is routinely used to inform clinical management. Specifically, the progression rate from low‐grade dysplasia (LGD) to high‐grade dysplasia (HGD)/EAC ranges from 0.5 to 13.0% per year.^5^, ^6^, ^7^ This wide range of progression rates may be attributed to the subjectivity of the histopathological diagnosis of LGD. In routine practice this often leads to overdiagnosis, particularly in community hospitals.^8^ Several studies have demonstrated that the interobserver agreement (IOA) rate for a diagnosis of LGD varies from poor to fair.^6^, ^9^, ^10^ A consensus diagnosis by multiple pathologists leads to a more robust LGD diagnosis, which also correlates with a significantly higher risk of progression.^7^, ^9^, ^11^ By contrast, high‐grade dysplasia has higher IOA and relates to a progression rate to EAC of approximately 20% per year.^12^

Indefinite for dysplasia in BE (BE‐IND) is diagnosed when the degree of cellular atypia is suggestive but not definite for dysplasia. The risk for BE‐IND progression is higher than non‐dysplastic BE (NDBE) and quoted in approximately 1.5 cases per 100 person‐years.^13^, ^14^ However, the IOA for BE‐IND is poor and overall lower than that of LGD.^15^, ^16^ This is partly because cellular atypia may be present within the background of excess inflammation^17^ or technical artefact.^18^ Therefore, diagnostic adjuncts are required to improve the reliability of this diagnosis.

Immunohistochemical staining for the tumour suppressor protein p53 (p53‐IHC) is a clinically applicable candidate marker. Evidence shows that aberrant p53 expression correlates with an increased risk of both incident and prevalent dysplasia.^19^, ^20^, ^21^ In particular, in patients with BE‐IND aberrant p53 expression was related to a fourfold increase in HGD/cancer incidence.^22^ Moreover, there is evidence that p53‐IHC may be used as a diagnostic adjunct to assist pathologists when dysplasia is suspected. For example, Kaye *et al*. demonstrated that p53‐IHC improved IOA for dysplasia among 10 pathologists with no previous experience in p53‐IHC.^23^ In this study, 72 cases with the full spectrum of BE grades were assessed, including BE‐IND.^23^ Similarly, in another study including 10 dedicated GI pathologists assessing 60 slides (two‐thirds with definite dysplasia and one‐third non‐dysplastic), p53‐IHC improved the IOA from 0.45 to 0.57 and slightly reduced the rate of BE‐IND diagnosis.^24^ In addition, there is evidence that p53‐IHC reduces by 6% the rate of major diagnostic errors (e.g. NDBE overinterpreted as either LGD or HGD) by pathologists with varying degrees of experience in BE assessment.^16^ Lastly, recent evidence shows that the P53‐IHC status, among other clinical markers, was found to be a useful adjunct to the non‐endoscopic sampling device (Cytosponge) in identifying high‐risk patients to prioritize for endoscopic evaluation.^25^

With this background, we set out to investigate the utility of p53‐IHC in the assessment of BE‐IND cases. We established a large multicentre cohort of samples with a previous BE‐IND diagnosis and evaluated the IOA and the rate of confirmed BE‐IND diagnosis by multiple pathologists.

## Materials and methods

### DATA COLLECTION

In this multicentre retrospective cohort study, we have analysed endoscopic tissue samples originating from patients with BE‐IND diagnosis from two academic referral centres in the United Kingdom (Cambridge University Hospital, Cambridge, UK) and the United States (Mayo Clinic, Rochester, MN, USA). Patients were identified within prospective research databases at each institution. At the time of screening, the Cambridge and Mayo Clinic databases included patients with BE diagnosis from 1999 to 2018 and 1997 to 2017, respectively. Inclusion criteria were: (i) age 18 years or older, (ii) endoscopic evidence of BE ≥1 cm in length; (iii) the presence of intestinal metaplasia (IM) on biopsies; and (iv) at least one biopsy from the study endoscopy was reported as BE‐IND. We excluded patients with a diagnosis of definite dysplasia of any grade either at a previous endoscopy or at the study endoscopy showing concomitant BE‐IND. All patients provided written, informed consent to be included in the research databases, and an ethics committee approval for this study was granted at both institutions (Cambridge University: LREC01/149; Mayo Clinic: 9–000514). All authors had access to the study data and reviewed and approved the final manuscript.

### HISTOLOGICAL ASSESSMENT – STAGE I [HAEMATOXYLIN AND EOSIN‐STAINED (H&E) ONLY]

The slides from eligible patients were reviewed by four expert GI pathologists. All pathologists involved in the study are experienced in assessing BE samples, each having a regular case volume of ~10–20 cases per week for 8–20 years. Pathologists were divided into two groups (groups A and B) to ensure that each sample was assessed independently by one pathologist from group A and one pathologist from group B. To avoid clustering in the assessment, pathologists from the two groups were paired randomly so that one pathologist could be compared with any of the two pathologists from the other group. In the first stage, only H&E slides were evaluated. The pathologists did not take part in the selection of study cases. Although some of the Cambridge study pathologists might have reported the original BE‐IND diagnosis, we did not look at the intraobserver diagnosis, as this was outside the scope of the study. The histological assessment was made using the Vienna classification.^17^ For the purpose of this study, basal crypt dysplasia was included in the definition of dysplasia.^26^ The established histopathological definition of BE‐IND included: (i) epithelial abnormalities that are insufficient to diagnose dysplasia of any grade – most commonly due to associated acute inflammation – and (ii) technical factors are precluding a reliable assessment of the epithelium, such as biopsy crushing artefact, thermal artefact and tangential embedding and sectioning. The study diagnosis was compared to the original diagnosis (BE‐IND) and was either confirmed or reclassified into:

- ‘no dysplasia’, when study pathologist‐diagnosed gastric or intestinal metaplasia without dysplasia, or
- ‘dysplasia’, when study pathologist diagnosed any degree of definite dysplasia [LGD, HGD, intramucosal cancer (IMC)].

The interobserver agreement (IOA) between pathologists in group A versus group B for each BE grade (NDBE, IND, LGD, HGD/IMC, all dysplasia) was calculated. All authors had access to the study data and reviewed and approved the final version of the manuscript.

### P53 IMMUNOCHEMISTRY STAINING

Sections from historical blocks corresponding to slides with the original diagnosis of BE‐IND were cut. Immunostaining was carried out on the Leica BOND‐MAX™ system using Bond polymer refined detection reagents (Leica Microsystems UK Ltd, Milton Keynes, UK) and a monoclonal antibody for wild‐type and mutant forms of p53, NCL‐L‐p53‐D07 (Leica Microsystems UK Ltd). The antibody was diluted at 1:50. Following staining, slides were counterstained using the Leica Autostainer XL and then cover‐slipped using the Leica fully automated glass coverslip.

### HISTOLOGICAL ASSESSMENT – STAGE II (H&E AND P53 IMMUNOSTAINING)

**Figure 1:**
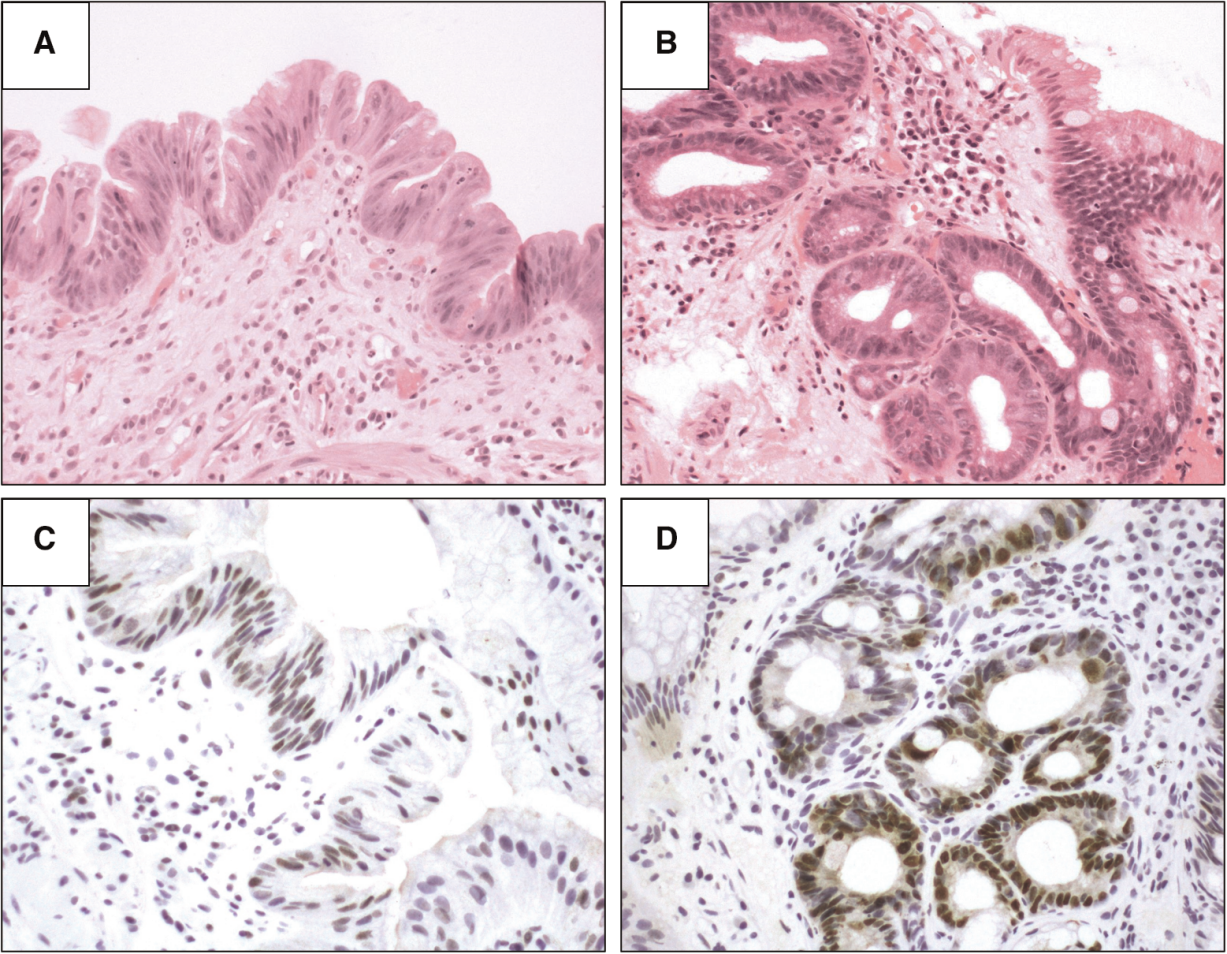
**A,** Barrett's oesophagus with features of indefinite for dysplasia (haematoxylin and eosin staining. **B,** Barrett's oesophagus with features of indefinite for dysplasia in basal crypts (haematoxylin and eosin staining). **C,** Same case as in **A** shows normal (wild‐type) p53 immunochemistry staining pattern. **D,** same case as in **B** aberrant (over‐expression) p53 immunochemistry staining pattern in basal crypts.

**Figure 2:**
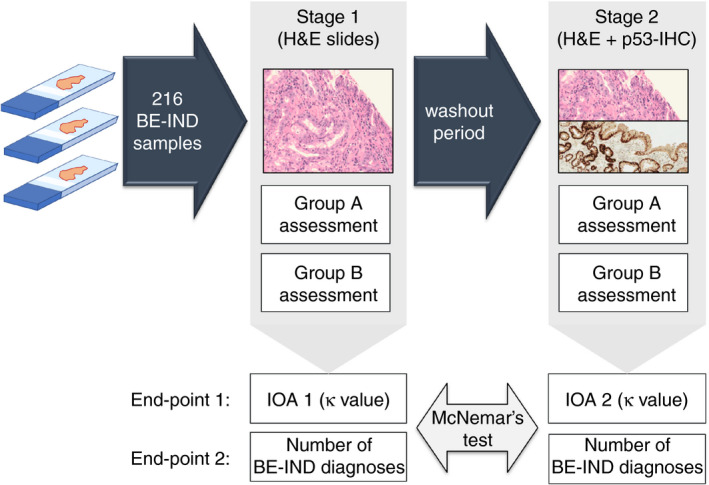
Design of the study. In stage I, each slide was assessed by one pathologist from group A and one pathologist from group B. After a wash‐out period of at least 8 weeks, the slides were assessed with matched p53‐stained slides available by the same pathologists as in stage I. In stage II, the pathologists were blinded to their original diagnosis. BE‐IND, Barrett's oesophagus indefinite for dysplasia; H&E, haematoxylin and eosin (staining); IOA, interobserver agreement. P53‐IHC, P53 immunohistochemistry.

Following a washout period of at least 8 weeks, each H&E slide was reviewed with matched p53‐IHC available by the same pathologists in groups A and B who assessed the samples in the first stage. The pathologists were blinded to their initial reading from the first stage of assessment. Aberrant p53‐IHC was regarded as either strong p53 staining compared to background level (overexpression) or focal absence of staining compared to background level (absent pattern), as illustrated in Figure 1. Normal p53 staining was regarded as uniform low or moderate staining intensity. In cases of uncertain staining patterns, the pathologist was allowed to report p53‐IHC status as equivocal. Similar to stage I, the rates of confirmed and reclassified diagnoses were calculated, and the IOA for each BE grade was generated. Additionally, the IOA for the p53‐IHC scoring was calculated. Figure 2 illustrates the study design.

### STATISTICS

Quantitative variables were described as means, medians with standard deviation (SD) and interquartile ranges (IQRs), where appropriate. Categorical variables were presented as counts and percentages of the cohort. The rates of changed diagnosis in the first and second histological assessment were compared using McNemar's test. The IOA for BE grades were generated using Cohen's kappa correlation coefficients (κ), with values ≤0 indicating no agreement, 0.01–0.20 no to poor agreement, 0.21–0.40 fair agreement, 0.41–0.60 moderate agreement, 0.61–0.80 substantial agreement and 0.81–1.00 almost perfect agreement. The κ values were generated with the ‘irr’ package (Gamer *et al*. 2019^27^) in R version 4.0.2 (R Foundation for Statistical Computing, Vienna, Austria).

The impact of p53‐IHC patterns on reclassifying the initial diagnosis (both to NDBE and dysplasia) was reported using odds ratios (ORs) calculated by median‐unbiased estimation (mid‐p) with 95% confidence intervals (CI). For all analyses, a *P*‐value of less than 0.05 was considered statistically significant. This report was written following the Strengthening the Reporting of Observational Studies in Epidemiology (STROBE) statement for cohort studies.^28^

### ROLE OF FUNDING SOURCE

No funders had any role in study design, data collection, data analyses, interpretation or writing of the report.

## Results

### COHORT CHARACTERISTICS

A total of 216 BE‐IND samples from 185 patients (Cambridge *n* = 65, 35.1%; Mayo Clinic *n* = 120, 64.9%) were included in the study (mean age = 64.8 ± 11.6 years, 155 males = 83.8%). The median BE length was 4 cm (IQR = 1–7 cm). Forty‐two patients had evidence of multifocal IND (22.7%) and 63 patients had persistent IND during the follow‐up (FU; 34.1%). The median FU time was 5.3 years (IQR = 1.3–9.2 years).

At the time of the BE‐IND diagnosis, 75 samples (34.7%) had no features of background inflammation and 132 samples (61.1%) had evidence of inflammation (98 samples with mild and 34 with moderate inflammation). In most cases, the diagnosis of BE‐IND was made due to background inflammation (*n* = 113; 52.3%) and cellular atypia of unknown significance (*n* = 95; 44.0%), and less often due to technical artefact (*n* = 3; 1.4%). Cohort characteristics are presented in Table 1.

**Table 1:** Cohort characteristics

Patient characteristics	
Cohort, no. (%)	185
Cambridge University	65 (35.1%)
Mayo Clinic	120 (64.9%)
Male sex, no. (%)	155 (83.3%)
Age, mean (± SD); years	64.8 (± 11.6)
Barrett's length (maximum extent); median (IQR); centimetres	4 (1–7)
Multifocal IND, no. (%)	42 (22.7%)
Persistent IND, no. (%)	63 (34.1%)
Presence of hiatus hernia, no. (%)	145 (78.4%)
Smoking status, no. (%)	
Never	65 (35.1%)
Former	71 (38.4%)
Active	13 (7.0%)
Unknown	36 (19.5%)
Sample characteristics	
Total number, no.	216
Cambridge University	96 (44.4%)
Mayo Clinic	120 (55.6%)
Background inflammation*, no. (%)	
None	75 (34.7%)
Mild	98 (45.4%)
Moderate	34 (15.7%)
Severe	0 (0.0%)
Missing data	9 (4.2%)
Cause of the BE‐IND diagnosis	
Inflammation	186 (76.9%)
Cellular atypia of unknown significance	43 (17.8%)
Technical artefact	7 (2.9%)
Unknown	6 (2.5%)

SD, standard deviation; IQR, interquartile range; BE‐IND; Barrett's oesophagus indefinite for dysplasia.

*Mild inflammation: occasional neutrophils within the surface epithelium or crypt epithelium or lamina propria; moderate inflammation: presence of crypt abscesses or scattered collections of neutrophils infiltrate within the surface epithelium; severe inflammation: presence of an ulceration.

### STAGE I: H&E SLIDES HISTOLOGICAL ASSESSMENT

**Figure 3:**
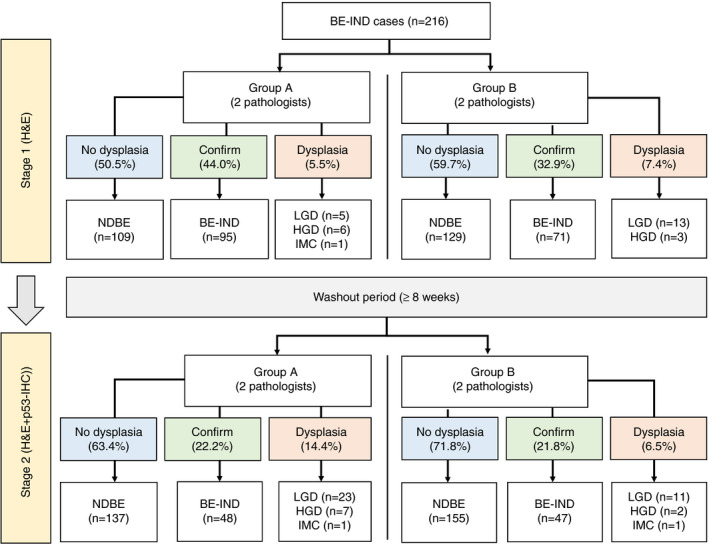
Flowchart showing the change in the diagnoses by the study pathologists in stage I [haematoxylin and eosin (H&E) only] and stage II (H&E + p53). BE‐IND, Barrett's oesophagus indefinite for dysplasia; HGD, high‐grade dysplasia; IMC, intramucosal cancer; LG, low‐grade dysplasia; NDBE, non‐dysplastic Barrett's oesophagus; P53‐IHC, P53 immunohistochemistry. [Colour figure can be viewed at wileyonlinelibrary.com]

In the first assessment, with H&E slides only, approximately half the cases were reclassified to NDBE, which occurred in 109 cases in group A (50.5%) and 129 cases in group B (59.7%) (Figure 3). The BE‐IND diagnosis was confirmed in 95 patients in group A (44.0%) and 71 patients in group B (32.9%). A reclassification to dysplasia was made in 12 patients (5.6%) for group A (five LGD, six HGD and one IMC) and in 16 patients (7.4%) for group B (13 LGD, three HGD).

The overall agreement among pathologists was only fair, with a κ = 0.23 (95% CI = 0.12–0.34) and a 57.4% agreement. On a BE grade level, the IOAs for NDBE, BE‐IND, LGD and HGD/IMC were 0.31 (95% CI = 0.19–0.44), 0.21 (95% CI = 0.07–0.34), −0.03 (95% CI = −0.50 to 0.43) and −0.02 (95% CI = −0.64 to 0.60), respectively. For all dysplastic cases, the IOA was 0.17 (95% CI = −0.17 to 0.51).

### STAGE II: H&E AND P53‐IHC SLIDES HISTOLOGICAL ASSESSMENT

Following at least 8 weeks' washout period, individual slides were reviewed by the same pathologists with p53‐IHC available (Figures 1 and 2). The p53‐IHC slides corresponding to 216 H&E BE‐IND diagnoses were assessed separately by the two pathologists from groups A and B.

In group A, 25 samples were scored as an aberrant p53‐IHC pattern (11.6%; three absent patterns and 22 overexpressions), 19 as equivocal (7.7%) and 141 as a normal pattern (65.3%). In group B, 16 samples were reported as an aberrant pattern (7.4%; two absent patterns and 14 overexpression), eight as equivocal (3.7%) and 168 as a normal pattern (77.8%). There was a substantial agreement between pathologists recognizing the p53‐IHC aberrant pattern with a κ value of 0.64 (95% CI = 0.47–0.82) and a percentage agreement of 92.9%.

**Figure 4:**
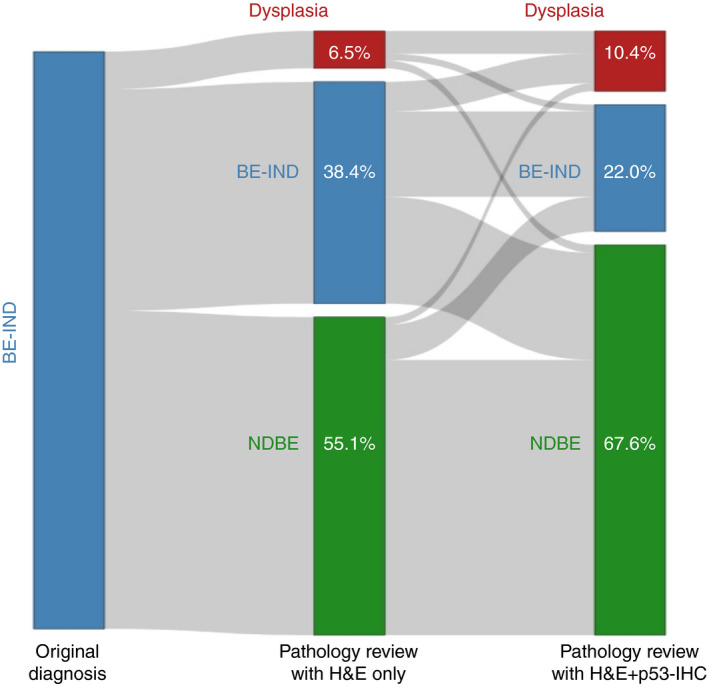
Sankey plot. BE‐IND, Barrett's oesophagus indefinite for dysplasia; H&E, haematoxylin and eosin (staining); NDBE, non‐dysplastic Barrett's oesophagus; P53‐IHC, P53 immunohistochemistry. Dysplasia category includes: low‐ and high‐grade dysplasia and intramucosal cancer. [Colour figure can be viewed at wileyonlinelibrary.com]

The fraction of cases reclassified to NDBE in stage II assessment increased to 63.4% (*n* = 137) in group A and 71.8% (*n* = 155) in group B (Figure 3). The overall number of NDBE diagnoses in groups A and B increased from 238 in stage I to 292 in stage II (*P* < 0.001). Conversely, the rate of confirmed BE‐IND cases decreased significantly to 48 cases (22.2%) and 47 cases (21.8%) in groups A and B, respectively (Figure 3). The overall number of BE‐IND diagnoses decreased from 166 (38.4%) to 95 (22.0%) within the two assessment stages (*P* < 0.001). Finally, a reclassification to definite dysplasia was made in 31 cases (14.4%; 23 LGD, seven HGD, one IMC) in group A and 14 cases (6.5%; 11 LGD, two HGD, one IMC) in group B. The total number of cases with dysplasia also increased significantly (18 in stage I versus 34 cases in stage II; *P* = 0.015), whereas the number of HGD/IMC remained similar, with 10 cases in stage I and 11 in stage II (*P* = 1.00). Figure 4 shows how slides were reclassified after reviewing with H&E only and with p53‐IHC available.

The overall agreement in the second stage of the assessment (H&E and p53‐IHC) increased from κ = 0.23 to κ = 0.39 (95% CI = 0.30–0.50), with a percentage agreement of 69.9%. The IOA for NDBE, BE‐IND, LGD and HGD/IMC increased to 0.46 (95% CI = 0.32–0.58), 0.26 (95% CI = 0.09–0.43), 0.49 (95% CI = 0.26–0.73) and 0.35 (95% CI = −0.13 to 0.83), respectively. Finally, for all dysplastic cases, the agreement increased to 0.44 (95% CI = 0.22–0.66). The changed IOA values are summarized in Table 2.

**Table 2:** The interobserver agreement among pathologists before and after p53 immunohistochemistry (stages I and II assessment)

The interobserver agreement (κ)	Stage I assessment (H&E)	Stage II assessment (H&E + p53‐IHC)
Overall	0.23 (95% CI = 0.12 to 0.34)	0.39 (95% CI = 0.30 to 0.50)
NDBE	0.31 (95% CI = 0.19 to 0.44)	0.46 (95% CI = 0.32 to 0.58)
BE‐IND	0.21 (95% CI = 0.07 to 0.34)	0.26 (95% CI = 0.09 to 0.43)
LGD	−0.03 (95%CI = –0.50 to 0.43)	0.49 (95% CI = 0.26 to 0.73)
HGD/IMC	−0.02 (95%CI = –0.64 to 0.60)	0.35 (95% CI = –0.13 to 0.83)
All dysplasia	0.17 (95% CI = –0.17 to 0.51)	0.44 (95% CI = 0.22 to 0.66)

BE‐IND, Barrett's oesophagus indefinite for dysplasia; H&E, haematoxylin and eosin (staining); HGD/IMC, high‐grade dysplasia/intramucosal cancer; LGD, low‐grade dysplasia; NDBE, non‐dysplastic Barrett's oesophagus; P53‐IHC, P53 immunohistochemistry.

### IMPACT OF THE P53‐IHC ON THE FINAL HISTOLOGICAL DIAGNOSIS

We then looked at how the p53‐IHC staining pattern impacted upon the subsequent grading of the initial BE‐IND diagnosis. We found a striking increase in the likelihood of a pathologist diagnosing definite dysplasia in the presence of an aberrant p53‐IHC pattern with an OR of 44.3 (95% CI = 18.8–113.0). Conversely, a non‐aberrant p53‐IHC pattern (normal/equivocal) was associated with an increased likelihood of NDBE diagnosis (OR = 18.0, 95% CI = 8.0–46.2).

Within the cases with aberrant p53 pattern (*n* = 19), most of the samples were reclassified to dysplasia both within groups A and B pathologists (63.2 and 52.6%, respectively). On the contrary, within the cases with non‐aberrant p53 pattern (*n* = 163), most of the samples were reclassified to NDBE (74.9% in group A and 81.0% in group B pathologists, respectively (see Supporting information, Figure S1).

## Discussion

In this study, we provide evidence that the addition of p53‐IHC reduces the rate of BE‐IND diagnosis and improves agreement among pathologists in the presence of cytological changes of difficult interpretation.

A diagnosis of BE‐IND is frequently encountered in clinical practice and represents between 3.6% and 8.4% of all BE diagnoses.^28^, ^29^ Excess of inflammation and atypia of unclear significance are the most common reasons behind BE‐IND diagnosis, followed by technical artefacts.^18^, ^29^ Although, by definition, BE‐IND lacks evidence of definite dysplasia, patients with this condition carry an increased risk of dysplasia compared to NDBE. Between 10% and 25% of BE‐IND patients will receive a neoplastic BE diagnosis within a year.^18^, ^30^, ^31^ Even though most BE‐IND patients will be reclassified to NDBE at follow‐up endoscopy, they remain at risk of incident neoplasia at long‐term FU. A recent meta‐analysis estimated the risk of incident HGD/EAC at approximately 1.5 per 100 person‐years, with a pooled incidence rate of LGD of 11.4 per 100 person‐years.^14^ For all these reasons, clinical guidelines recommend that in the absence of visible lesions, patients with BE‐IND should receive an increased dose of acid suppressant medication and repeat endoscopy at 6 months with mapping biopsies.^3^, ^4^, ^31^ This creates an additional burden to the endoscopy services and probably a degree of anxiety for the patients. Therefore, any effort to reduce the BE‐IND rate will positively impact the cost‐effectiveness of surveillance strategies and patient quality of life. Improved awareness of technical aspects and education on morphological features of definite dysplasia can help. There is evidence that the BE‐IND rate has nearly halved in a time‐span of approximately 10 years with a refinement of the histopathological diagnostic criteria for dysplasia.^29^ Our data clearly show that p53‐IHC is a valuable adjunct to assist the pathologist in interpreting the significance of cytological atypia, leading to a >40% reduction in BE‐IND diagnosis rate in our cohort. There remains, however, a proportion of cases where the addition of p53‐IHC does not lead to reclassification. This is important to note, as aberrant p53 status should not be interpreted as a surrogate of dysplasia and can be even found in cases of NDBE, and ultimately dysplasia is a morphological diagnosis.

The second most important caveat of BE‐IND diagnosis is the low level of IOA among pathologists. It is well established that a consensus diagnosis of dysplasia strengthens its clinical significance.^7^, ^9^, ^11^ This has been demonstrated for LGD, which is also problematic due to subjectivity in diagnosis. BE‐IND represents an even more contentious diagnosis, with very poor rates of IOA.^15^, ^16^ Only 33–44% of cases historically diagnosed with BE‐IND were confirmed after revision by the expert pathologists in our study. Most importantly, the overall κ value for IOA improved from 0.23 to 0.39 after the addition of p53‐IHC. The improvement in the IOA was particularly significant for dysplastic cases. Nevertheless, it was only marginal for confirmed BE‐IND, suggesting that this condition remains a subjective diagnosis despite all efforts. However, an improved overall agreement for dysplasia diagnosis carries a significant clinical benefit, as it can streamline management decisions and trigger more timely therapeutic intervention. Because of recent advances in computer‐based image analysis to digitized pathology slides, p53‐IHC may be an attractive target for machine learning. Algorithms able to accurately differentiate aberrant p53‐IHC slides would be of great value in a clinical setting, reducing the pathologists' burden. It needs to be emphasized that our data do not necessarily support the use of p53‐IHC in routine reporting of straightforward non‐dysplastic biopsies. However, this might help in the future for risk stratification in selected cases.

This study has several strengths. We have studied a large cohort of patients with BE‐IND from two high‐volume centres with geographical diversity, which helps to draw a clear conclusion on the role of p53‐IHC in this subgroup of patients. Although previous studies had demonstrated the added benefit of p53‐IHC in BE, BE‐IND cases were either absent or sparsely represented in these cohorts. We have used a robust methodology with four expert GI pathologists randomly paired, which allowed us to calculate the IOA of different pairs of observers. We chose this design as the number of cases was high, and each slide's assessment by all four pathologists would be unfeasible. In other studies, a larger group of pathologists (*n* = 10–51) reviewed smaller cohorts of cases (*n* = 6–70).^16^, ^23^, ^24^

This study has a few limitations. The four expert pathologists involved in this study work in the same institution; therefore, the results might not be generalized to a broader community of pathologists with different degrees of experience. However, clinical guidelines recommend expert pathologist review in case of suspected dysplasia; therefore, BE‐IND should be reviewed by a pathologist with interest and experience in BE diagnosis. When the study was conceived digital pathology was not widely available, and a decision to have pathologists from a single institution had to be taken. In addition, criteria for BE‐IND diagnosis might have changed over time, although this is difficult to characterize. For this reason, some of the historical cases included in this study might not fully represent biopsies currently reported as BE‐IND; however, we felt inclusion for historical cases was required to build a sufficiently large cohort to address the study objective with statistical power.

In summary, we provide evidence that p53‐IHC has a robust clinical utility to help the pathologist report cases with atypical cytological changes, with and without definite dysplasia features, and should be routinely used, particularly when BE‐IND diagnosis is considered.

## Conflicts of interest

All the authors disclose no conflicts of interest.

## Author contributions

W Januszewicz: concept and design of the study, data analysis and interpretation, drafting and revising the manuscript; N D Pilonis: acquisition of data, drafting and revising the manuscript. T Sawas: acquisition of data, drafting and revising the manuscript; R Phillips: acquisition of data, drafting and revising the manuscript; M O'Donovan: histopathological assessment of the samples, analysis and interpretation of data, drafting and revising the manuscript; A Miremadi: histopathological assessment of the samples, analysis and interpretation of data, drafting and revising the manuscript; S Malhotra: histopathological assessment of the samples, analysis and interpretation of data, drafting and revising the manuscript; M Tripathi: histopathological assessment of the samples, analysis and interpretation of data, drafting and revising the manuscript; A Blasko: p53‐IHC staining and interpretation, acquisition of data, analysis and interpretation of data, drafting and revising the manuscript; D A Katzka: concept and design of the study, drafting and revising the manuscript; R C Fitzgerald: concept and design of the study, data analysis and interpretation, drafting and revising the manuscript; M di Pietro: concept and design of the study, data analysis and interpretation, drafting and revising the manuscript; all authors revised and accepted the final version of the manuscript.

## Supporting information


**Figure S1.** Sankey plot representing the impact of p53 immunostaining patterns in reclassifying the original BE‐IND diagnosis separately in Group A and Group B pathologists. BE‐IND; Barrett's oesophagus indefinite for dysplasia, NDBE; non‐dysplastic Barrett's oesophagus.

## Data Availability

Data available on request due to privacy/ethical restrictions.
