# Supplementary figures and images for: The utility of P53 immunohistochemistry in the diagnosis of Barrett's oesophagus with indefinite for dysplasia
Source: Histopathology. 2022 May 20;80(7):1081–90. doi: 10.1111/his.14642 (PMC9321087; doi:10.1111/his.14642)

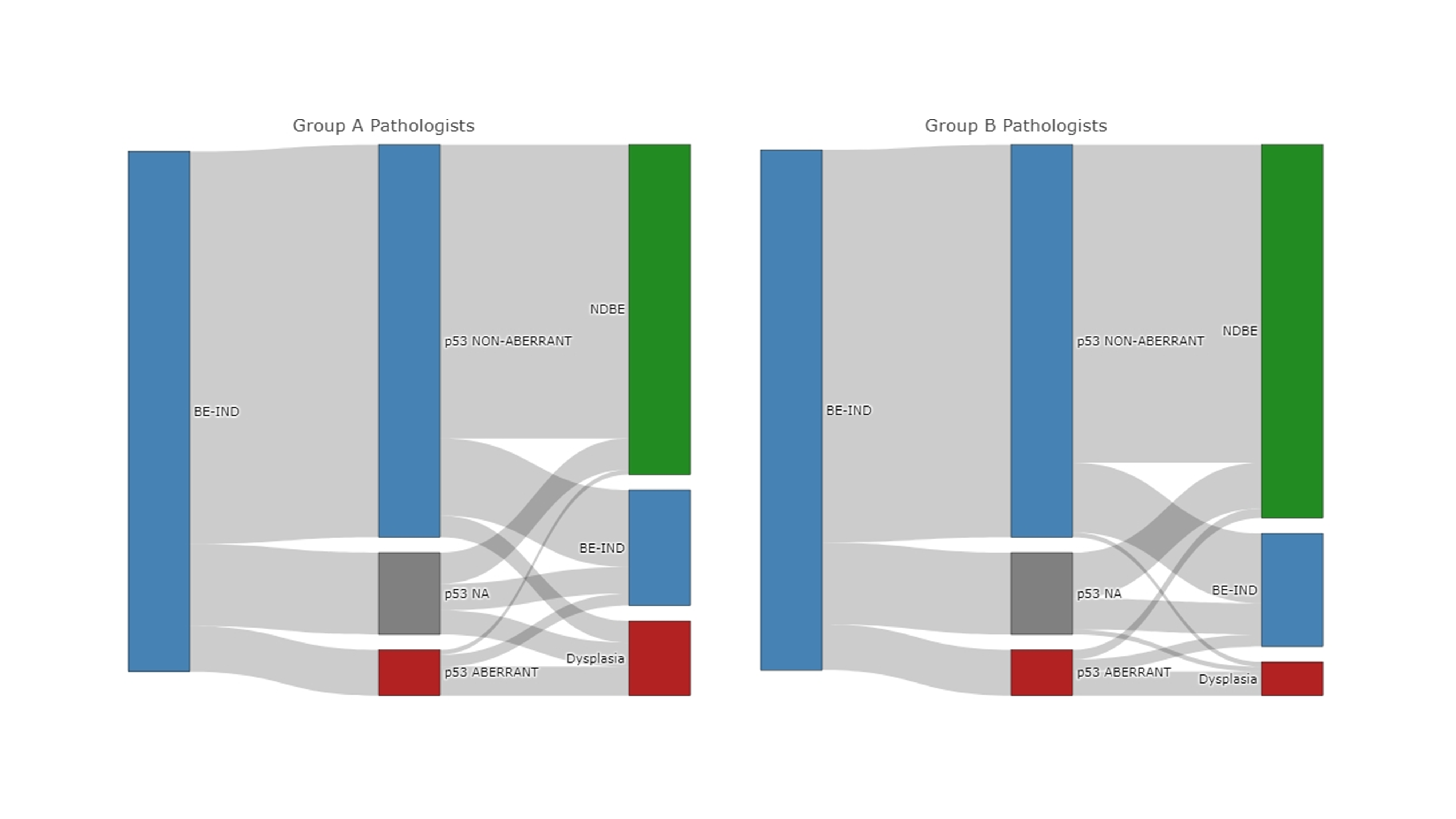

Supplement: Supplementary file 1 — Figure S1. Sankey plot representing the impact of p53 immunostaining patterns in reclassifying the original BE‐IND diagnosis separately in Group A and Group B pathologists. BE‐IND; Barrett's oesophagus indefinite for dysplasia, NDBE; non‐dysplastic Barrett's oesophagus. [file HIS-80-1081-s001.tif]
